# 1469. Development and Implementation of a Predictive Model for a Semi-automated Surgical Site Infection Surveillance System

**DOI:** 10.1093/ofid/ofad500.1306

**Published:** 2023-11-27

**Authors:** Sojeong Lee, Jiyeon Joo, Miseo Kim, Seung Hee Ryu, Jeong Young Lee, Hyejin Yang, So-Yeon Park, Eun Ok Kim, Jiwon Jung, Sung-Han Kim

**Affiliations:** Asan Medical Center, Seoul, Seoul-t'ukpyolsi, Republic of Korea; Asan Medical Center, Seoul, Seoul-t'ukpyolsi, Republic of Korea; Asan Medical Center, Seoul, Seoul-t'ukpyolsi, Republic of Korea; Asan Medical Center, Seoul, Seoul-t'ukpyolsi, Republic of Korea; Asan Medical Center, Seoul, Seoul-t'ukpyolsi, Republic of Korea; Asan Medical Center, Seoul, Seoul-t'ukpyolsi, Republic of Korea; Asan Medical Center, Seoul, Seoul-t'ukpyolsi, Republic of Korea; Asan medical center, Songpa-gu, Seoul-t'ukpyolsi, Republic of Korea; Asan Medical Center, Seoul, Seoul-t'ukpyolsi, Republic of Korea; Asan medical center, Songpa-gu, Seoul-t'ukpyolsi, Republic of Korea

## Abstract

**Background:**

Automated surveillance systems for healthcare-associated infections have been implemented worldwide, but monitoring surgical site infections (SSI) is still challenging. Surveillance of SSI requires a review of multiple clinical aspects and medical records, which is time-consuming and prone to human error. We aimed to develop a predictive model for SSI and implement a semi-automated surveillance system.

**Methods:**

The study included 213 major surgeries randomly selected from 25 different types at a tertiary care hospital in Seoul, South Korea, between June 2019 and May 2022. Infection preventionists diagnosed SSI based on US National Healthcare Safety Network criteria. After identifying variables significantly associated with SSI through univariate analysis, we developed several predictive models. We then conducted multivariable logistic regression analysis, calculated coefficients and p-values, and evaluated model performance using Area Under the ROC Curve (AUC) analysis, as well as sensitivity, specificity, positive predictive value (PPV), and negative predictive value (NPV).

**Results:**

Of 213 surgeries, 13 (6.1%) were exploratory laparotomy, while 11 (5.2%) were cesarean section and ventricular shunting each. 74 cases (34.7%) were diagnosed with SSI. The study found that a predictive model that included use of antimicrobial agent, positive culture test, infection-related imaging findings (e.g., CT, MRI, ultrasonography), record of wound dressing, and pus or dehiscence of wound records, identified patients at high risk for SSI with an AUC of 0.853 (95% CI: 0.795-0.911). We weighted these variables and found that with a cutoff of 4 or higher out of a total of 8 points demonstrated sensitivity of 75.7% (95% CI: 65.9-85.5), specificity of 82.7% (95% CI: 76.5-89.0), PPV of 86.5% (95% CI: 80.7-92.3), and NPV of 80.3% (95% CI: 74.9-85.6). Based on this model, a semi-automated surveillance system was developed.

**Conclusion:**

The predictive model and semi-automated surveillance system of SSI have the potential to improve the efficiency and accuracy. Further optimization of the predictive model by validating it for different types of surgeries and incorporating additional infectious variables and weights for each variable to increase its accuracy and applicability is needed.

**Disclosures:**

**All Authors**: No reported disclosures

